# The Effect of Fish Oil-Based Lipid Emulsion and Soybean Oil-Based Lipid Emulsion on Cholestasis Associated with Long-Term Parenteral Nutrition in Premature Infants

**DOI:** 10.1155/2016/4139164

**Published:** 2016-03-24

**Authors:** Leilei Wang, Jing Zhang, Jiejin Gao, Yan Qian, Ya Ling

**Affiliations:** ^1^Department of Pediatrics, The First Affiliated Hospital, Wenzhou Medical University, Wenzhou 325000, China; ^2^The Second Clinical Medical College, Wenzhou Medical University, Wenzhou 325000, China

## Abstract

*Purpose*. To retrospectively study the effect of fish oil-based lipid emulsion and soybean oil-based lipid emulsion on cholestasis associated with long-term parenteral nutrition in premature infants.* Methods*. Soybean oil-based lipid emulsion and fish oil-based lipid emulsion had been applied in our neonatology department clinically between 2010 and 2014. There were 61 qualified premature infants included in this study and divided into two groups. Soybean oil group was made up of 32 premature infants, while fish oil group was made up of 29 premature infants. Analysis was made on the gender, feeding intolerance, infection history, birth weight, gestational age, duration of parenteral nutrition, total dosage of amino acid, age at which feeding began, usage of lipid emulsions, and incidence of cholestasis between the two groups.* Results*. There were no statistical differences in terms of gender, feeding intolerance, infection history, birth weight, gestational age, duration of parenteral nutrition, total dosage of amino acid, and age at which feeding began. Besides, total incidence of cholestasis was 21.3%, and the days of life of occurrence of cholestasis were 53 ± 5.0 days. Incidence of cholestasis had no statistical difference in the two groups.* Conclusion*. This study did not find the different role of fish oil-based lipid emulsions and soybean oil-based lipid emulsions in cholestasis associated with long-term parenteral nutrition in premature infants.

## 1. Introduction

Parenteral nutrition related cholestasis (PNAC) is the most serious complication of the long-term parenteral nutrition (PN). Nevertheless, its pathogenesis still remains unclear. Some studies have shown that [1–3] omega-3 fish oil fatty acid is helpful to the recovery of cholestasis in infants. Our neonatology department has applied fish oil-based lipid emulsion and soybean oil-based lipid emulsion since 2010. This study retrospectively investigated the role of soybean oil-based lipid emulsion and fish oil-based lipid emulsion in cholestasis associated with long-term parenteral nutrition in premature infants who were admitted to our hospital during 2010–2014.

## 2. Materials and Methods

### 2.1. Patients and Enrollment Criteria

We conducted a retrospective chart review of all neonates who were admitted to the neonatal intensive care unit at The First Affiliated Hospital of Wenzhou Medical University and received PN for more than 14 days between 2010 and 2014, following the approval of the Regional Ethics Committee of The First Affiliated Hospital of Wenzhou Medical University. Informed consent was harvested from parents at recruitment. Premature infants with death consequences, abnormal metabolic disease, cardiac failure, bowel obstruction, sepsis, urinary tract infection, hypothyroid and congenital heart disease, or history of using antifungal drug during hospitalization were excluded from this study. TORCH (*Toxoplasma gondii*, other agents,* Rubella*,* Cytomegalovirus*, and Herpes Simplex Virus) examination and abdominal B-ultrasound examination in qualified premature infants were performed to exclude cholestasis caused by other factors such as biliary obstruction and viral infection.

### 2.2. Parenteral Nutritional Support

Parenteral nutrition formulations include glucose, amino acid compound for children, lipid emulsions, water-soluble vitamins, fat-soluble vitamins, and electrolytes. The initial amount of amino acids product was 1.0 g/(kg·d) and gradually increased by 0.5 g/(kg·d) up to 3.0 g/(kg·d). The initial amount of lipid emulsions product was 1.0 g/(kg·d) and gradually increased by 0.5 g/(kg·d) up to 3.0 g/(kg·d). Glucose started from the initial 6–8 g/(kg·d) and gradually increased according to the glucose tolerance, but no more than 12–16 g/(kg·d). Continuous uniform infusion of parenteral nutrition with minipump via peripheral or central venous lasted 24 hours.

### 2.3. Lipid Emulsion Administration in Neonates

Two kinds of lipid emulsions had been applied in our neonatology department clinically between 2010 and 2014. They were Baxter-Kalu (Guangzhou Baxter Qiaoguang Healthcare Co. Ltd.) and Omegaven (Fresenius Kabi Austria GmbH). The Baxter-Kalu was 20% (20 g/dL) lipid (50% soy, 50% medium chain triglycerides). The Omegaven was 10% (10 g/dL) lipid (100% fish oil). *ω*-6 fatty acid was the main component of soybean oil, while *ω*-3 fatty acid was the main component of fish oil. In this study, premature infants were divided into two groups according to different clinical parenteral nutrition lipid emulsions treatment: soybean oil group and fish oil group. During the course of the research, blood liver function of premature infants was regularly tested to monitor the occurrence of cholestasis. In addition, the infants who could receive enteral nutrition were fed on Wyeth premature infant milk powder. The initial volume of enteral feeding is 10–15 mL/(kg·d), aiming to reach full enteral feeding of 150–180 mL/(kg·d).

### 2.4. Diagnostic Criteria on Parenteral Nutrition Associated Cholestasis

Parenteral nutrition was kept for at least 14 days; the main symptoms and signs in clinic were skin jaundice and (or) lighter stool color and serum direct bilirubin (DB) >34 *μ*mol/L (2 mg/dL) [4].

### 2.5. Diagnostic Criteria on Feeding Intolerance

Feeding intolerance in the premature infant is the inability to digest enteral feedings presented as gastric retention, abdominal distension, and necrotizing enterocolitis prodrome.

### 2.6. Statistical Methods

Continuous variables with normal distribution were presented as mean ± standard deviation, while categorical variables were presented as number (percentage). Between-group comparisons of enumeration data were performed using chi-squared test as appropriate. Independent sample *t*-test was used to compare the measurement data normally distributed between soybean oil group and fish oil group. Two-tailed test was used. *P* values <0.05 were considered statistically significant.

## 3. Results

### 3.1. Comparison of Basic Demographics

Only 61 newborns met the study criteria and were included in this study. Soybean oil group was made up of 32 premature infants, while fish oil group was made up of 29 premature infants. Chi-square test analysis showed that there were no differences in terms of gender, feeding intolerance, and the incidence of infection history in the two groups (Table 1). *t*-test analysis between the two groups showed no difference in birth weight, gestational age, age at which feeding began, or the days of life of occurrence of cholestasis (Table 1). And no significant difference was found in the administration of intravenous nutrition in the two groups (Table 2), indicating good comparability between the two groups.

### 3.2. Comparison of the Incidences of Cholestasis

In this study, total incidence of cholestasis was 21.3%, and the days of life of occurrence of cholestasis were 53.0 ± 17.9 days. The incidence of cholestasis was 21.9% (7/32) in the soybean oil group as compared to 20.7% (6/29) in the fish oil group (*P* = 0.910, Table 3), which showed no significant difference between them.

## 4. Discussion

Neonatal parenteral nutrition associated cholestasis nowadays has attracted extensive attention from the pediatricians and neonatologists. A large number of clinical studies have found that risk factors for developing PN-induced cholestasis include small gestational age, low birth weight, infection history, absolute diet fasting, duration of PN, dosage of amino acids, and dosage of lipids in PN [5–7]. For example, Goulet et al. have confirmed the relevance between cholestasis and intravenous infusion of lipid emulsion [8]. Cholestasis in adult is associated with the amount of parenteral nutrition, while cholestasis in children is related to the lipid types and management style [1, 8, 9]. It is important to note that excess energy provided by lipid emulsion (lipid emulsion overload) has the potential to lead to liver steatosis and rise of blood lipids [8]. However, lipid emulsion, which provides high energy density, is also an indispensable component in neonatal parenteral nutrition. In particular, lipid emulsions can provide essential fatty acids for newborns who have not received intestinal feeding yet, that is, the explanation for the increasing common interests and expanded research scope in intravenous lipid emulsions in the neonatal intensive care in recent years [10].

PNAC tends to occur after two-week administration of parenteral nutrition. Chinese scholars, Wang et al. [4], reported that premature infants suffered from PNAC after 3.3 ± 1.6 weeks of administration of parenteral nutrition. And Taiwan scholars demonstrated that cholestasis associated with long-term parenteral nutrition in premature infants appeared for 57.5 ± 25.8 days after birth [11]. In this study, it was found that premature PNAC occurred for 53 ± 5.0 days after parenteral nutrition. In addition, the incidence of cholestasis was 21.3% in this study, while Chinese reports declared that incidence of PNAC in premature infants or newborns with low/extremely low birth weight ranged from 8.8% to 20.9% [12, 13]. Other literature has also reported that incidence of parenteral nutrition related cholestasis was 20.7% [6].

As indicated by previous studies [1–3], fish oil-based lipid emulsions can protect the liver cells for they are rich in omega-3 composition, so as to improve the clinical prognosis for PNAC children, while soybean oil-based lipid emulsions have the potential to reduce the secretion of bile and cause liver damage for they are rich in omega-6 composition. Therefore, the relationship between fish oil and PNAC becomes a research direction currently. This study retrospectively investigated the role of different lipid emulsions in cholestasis associated with long-term parenteral nutrition in premature infants who were admitted to our hospital during 2010–2014. According to the statistical analysis, basic data of two groups had no statistical difference, suggesting good comparability in terms of the influence of different lipid emulsions types on cholestasis. In this study, we found that the influence of different lipid emulsions on cholestasis had no statistical difference. Other studies [1–3] showed that fish oil-based lipid emulsions are beneficial to improving the liver function and reducing cholestasis after the occurrence of cholestasis in children. Nevertheless, the focus of our study was to analyze whether there was a significant difference on preventing cholestasis between soybean oil-based lipid emulsions and fish oil-based lipid emulsions in premature infants that need long-term parenteral nutrition before the occurrence of cholestasis. Our results showed that there was no protection from cholestasis of omega-3 based lipids.

According to* Clinical Guidelines For Neonatal Nutrition Support in China*, the calculation formula of average calories provided by parenteral nutrition for premature infants is PN = (1 − EN/110) × 80. PN means parenteral nutrition, and EN means enteral nutrition. Recommended calorie intake is 110 kcal·kg^−1^·d^−1^ via the full enteral feeding and 80 kcal·kg^−1^·d^−1^ via the full parenteral feeding.

Limitation of our study included the small number of cases and the lack of both complete information and long-term follow-up data in the retrospective analysis, which decrease the credibility of analysis results inevitably.

The etiology of parenteral nutrition associated liver disease still remains unclear and is thought to be multifactorial. This study did not find the different role of fish oil-based lipid emulsions and soybean oil-based lipid emulsion in cholestasis associated with long-term parenteral nutrition in premature infants.

## Figures and Tables

**Table 1:** Demographics of premature infants.

	Soybean oil group (*n* = 32)	Fish oil group (*n* = 29)	*χ* ^2^/*t* value	*P* value
Gender: male (%)	22 (68.8%)	18 (62.1%)	0.301	0.583
Feeding intolerance (%)	8 (25.0%)	10 (34.5%)	0.658	0.417
Infection history (%)	22 (68.8%)	19 (65.5%)	0.072	0.788
Birth weight (g)	1119.8 ± 134.1	1123.7 ± 113.5	0.777	0.382
Gestational age (week)	28.8 ± 1.3	28.9 ± 1.4	0.188	0.666
Age at which feeding began (day)	8.1 ± 4.6	10.9 ± 6.9	2.214	0.142

Continuous variables with normal distribution were presented as mean ± standard deviation, while categorical variables were presented as number (percentage). Between-group comparisons of enumeration data were performed using chi-squared test as appropriate. Independent sample *t* test was used to compare the measurement data normally distributed.

**Table 2:** Composition and duration of PN.

	Soybean oil group (*n* = 32)	Fish oil group (*n* = 29)	*t* value	*P* value
Total dosage of lip (g)	23.8 ± 17.8	24.3 ± 24.2	2.933	0.092
Duration of lip (d)	31.8 ± 13.2	33.5 ± 15.0	1.150	0.288
Duration of PN (d)	35.5 ± 13.5	43.8 ± 15.2	0.730	0.396
Total dosage of aa (g)	74.8 ± 43.4	78.6 ± 50.4	0.521	0.473

Lip: lipids; aa: amino acids; PN: parenteral nutrition.

**Table 3:** Comparison the incidences of cholestasis.

	Cholestasis		*χ* ^2^	*P* value
	No	Yes		
Soybean oil group (*n* = 32)	25 (78.1%)	7 (21.9%)	0.013	0.910
Fish oil group (*n* = 29)	23 (79.3%)	6 (20.7%)		
